# Joint-preserving regenerative therapy for patients with early-stage osteonecrosis of the femoral head

**DOI:** 10.1186/s41232-016-0002-9

**Published:** 2016-04-25

**Authors:** Yutaka Kuroda, Shuichi Matsuda, Haruhiko Akiyama

**Affiliations:** 1 Department of Orthopaedic Surgery, Graduate School of Medicine, Kyoto University, Shogoin, Kawahara-cho 54, Sakyo-ku, Kyoto 606-8507 Japan; 2 Department of Orthopaedic Surgery, Gifu University, Gifu, Japan

**Keywords:** Osteonecrosis, Femoral head, Regenerative therapy, Growth factor, Fibroblast growth factor, Clinical trial

## Abstract

Osteonecrosis of the femoral head is an intractable disease often occurring in patients aged 30–40 years that can cause femoral head collapse, pain, and gait disturbance. Background factors, including corticosteroid use, alcohol intake, and idiopathic causes, have been indicated. It is estimated that 70–80 % of osteonecrosis patients experience femoral head collapse, for which total hip arthroplasty is considered the most effective treatment, even in young patients. Thus, there is a crucial need for developing a minimally invasive regenerative therapy as a preventive surgery for femoral head collapse: this has been an important area of research in the past decades. Core decompression, the most popular minimally invasive surgery for osteonecrosis of the femoral head, has been used for a long time; however, it has been insufficient to prevent femoral head collapse. For further improvement in therapeutic efficacy, cell transplantation and the use of artificial bone and growth factors have been proposed in addition to core decompression. Since 2000, newer therapies such as autologous bone marrow cell transplantation and the embedding of metal implant rods have been developed in Europe and the USA; however, these approaches have yet to become a global standard. This practical review summarizes applied state-of-the-art regenerative therapy-based core decompression. We introduce the clinical application of recombinant human fibroblast growth factor (rhFGF)-2-impregnated gelatin hydrogel for patients with precollapse osteonecrosis of the femoral head. Radiography and computed tomography have confirmed bone regeneration inside the femoral heads around the region of rhFGF-2 gelatin hydrogel administration. With further development, the minimally invasive method, which can be expected to promote bone regeneration in necrotic areas, could become a useful early-stage treatment for osteonecrosis of the femoral head. Patients can resume their daily routine soon after surgery, and the procedure is inexpensive. As such, it is a promising regenerative therapy that can be actively employed in osteonecrosis of the femoral head before femoral head collapse.

## Background

Osteonecrosis of the femoral head (ONFH) is a destructive disease of the hip joint caused by a critical decrease in the vascular supply to the femoral head. Several causative factors have been indicated, including corticosteroid use, alcohol intake, hypercoagulation, bone marrow fat embolisms, elevation of intraosseous pressure in the femoral head, and vascular endothelial dysfunction [1–4]. However, the pathogenesis of ONFH is poorly understood. ONFH often occurs in young adults who are in their 30s, and it occurs bilaterally in approximately half of the cases. Steroid-induced ONFH is commonly encountered by orthopedic surgeons and in other medical departments (such as collagen disease, rheumatology, hematology, nephrology, transplant surgery, respiratory, dermatology, and ophthalmology departments) where steroid pulse therapies are performed. Thus, magnetic resonance imaging (MRI) can be performed for early diagnosis, especially for patients receiving steroid pulse therapy.

Core decompression, which is frequently used in Europe and the USA [2, 3, 5], is a minimally invasive surgery; however, if the necrotic area is large, the effect of core decompression may be limited, and femoral head collapse often occurs regardless. This procedure is almost never performed in Japan, except for biopsy purposes. Other conventional joint-preserving therapies, such as trochanteric rotational osteotomy (the most common hip joint surgery in Japan) and vascularized bone grafts, are difficult, highly invasive, and entail several months of recovery before the patient can resume a daily routine. Considering the age at which ONFH commonly occurs, opting for such operations while the patient is still asymptomatic is difficult. Both the patients and surgeons have difficulty in deciding on a method and the timing of these operations.

Femoral head collapse occurs in 70–80 % of ONFH cases, depending on the size and location [6, 7]. After femoral head collapse, the ONFH site develops secondary osteoarthritis (OA), destroying both the femoral head and the acetabulum [2, 3, 8]. Total hip arthroplasty (THA) is considered an effective treatment for secondary osteoarthritis, even in young patients [3, 7]. As THA is invasive and requires a revision surgery, it is crucial to develop a minimally invasive regenerative therapy that can preserve the femoral head, thus preventing collapse. This has been a research focus for many years [2, 3, 9–11].

Cell therapy [12–16], proteins, and other bone substitutes [9, 17, 18] have been proposed, and various types of cell therapies using autologous marrow cells or stem cells are already being attempted, though they have not yet become standardized. Non-cellular therapeutic strategies using growth factors have also been proposed; however, verification in animal experiments has made little progress, primarily because of the absence of an animal model for femoral head-specific necrosis [9, 19, 20] and secondarily because of the lack of a technique to locally deliver the growth factor [21].

To help address this problem, we reported a new rabbit model in which early ONFH progresses to femoral head collapse and OA, similar to that in humans. While animal models of corticosteroid treatment alone do not develop the characteristics of advanced ONFH seen in humans [22], we applied a rabbit model of ONFH induced by a combination of methylprednisolone administration and vascular occlusion of the capital femoral epiphysis by electrocoagulation. The rabbits started to develop ONFH around 4 weeks after the ONFH procedure and established ONFH within 8 weeks [23]. In this model, we showed that a single local injection of recombinant human fibroblast growth factor (rhFGF)-2-impregnated gelatin hydrogel, which has superior slow-release characteristics, suppresses the progression of femoral head necrosis. To translate this research to humans, the clinical application of controlled release rhFGF-2 for precollapse ONFH patients was performed at the Department of Orthopaedic Surgery in Kyoto University Hospital starting in March 2013 [24]. In this review article, we present the local application of rhFGF-2 in human ONFH and its clinical benefit compared to other treatments.

## Treatment strategy for ONFH

**Fig. 1:**
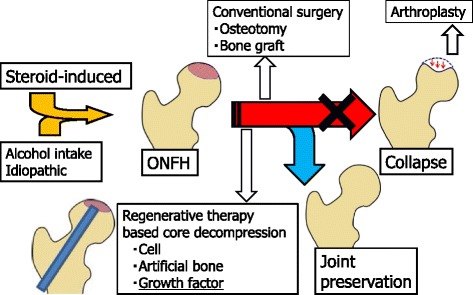
Treatment strategy for osteonecrosis of the femoral head. Scheme of treatment strategy for osteonecrosis of the femoral head (*ONFH*) is shown. In daily clinical cases, even if a patient is diagnosed with ONFH, most cases experience femoral head collapse without surgical treatments and finally have to undergo total hip arthroplasty. The ultimate goal for ONFH therapy is to prevent femoral head collapse. Surgical alternatives for preservation include osteotomy and vascularized bone grafting, but the procedures are difficult, technically demanding, and require long-term hospitalization. Therefore, there has been a great desire for a minimally invasive regenerative therapy that can prevent femoral head collapse. Several regenerative treatment options, including cell or stem cell transplantation, artificial bone substitutes, and administration of growth and differentiation factors, have been recently reported

Conventional surgical treatment, mainly core decompression, is used widely in Europe and the USA [2, 3, 5]; in contrast, in Japan, a variety of surgical osteotomy procedures have been performed. However, clinical results vary: the collapse rate of the femoral head was more than 70 % in core decompression with precollapse ONFH in one study [13], while others have reported a collapse rate of 50 % with rotational osteotomy [25]. A variety of surgical methods have also been reported, including free or vascularized methods with fibular and iliac bone grafts, allogeneic bone grafts, and bone cartilage transplantation (mosaicplasty); bone cement injection to the femoral head has also been reported, although it is not common. Currently, there is no consensus regarding the standard treatment (Fig. 1).

The use of MRI improves early diagnosis, especially in patients receiving steroid pulse therapy. Accordingly, the treatment target has emphasized aggressive early prevention of femoral head collapse. Core decompression alone is challenging [13, 26, 27]; therefore, cell transplantation and artificial bone or metal implants in the core site have also been attempted. In addition, a deliverable, growth factor-containing, bone-promoting substitute has also been proposed [2, 3, 9, 17, 18].

## Osteotomy

Over 40 years, various types of osteotomies have been performed for the treatment of ONFH. The concept of surgery consists of moving the necrotic region from the weight-bearing surface of the femoral head. These involve three-axis directions, varus-valgus, flexion-extension, and anterior-posterior rotation. These procedures are technically demanding and are popular in Asia, though less in other regions [3, 28].

## Bone grafting

Various surgical techniques have been reported for the use of bone grafts for the treatment of ONFH. In the 1930s, Phemister first reported a surgical procedure of a non-vascularized bone graft from the tibia for the treatment of ONFH [28]. Since then, newer techniques such as the lightbulb and trapdoor non-vascularized bone graft have been used with good results [29]. Another technique for bone grafting is vascularized option. Vascularized fibular graft was introduced in 1979 and consists of a fibular graft together with its vascular supply and harvested directly into the necrotic lesion of the femoral head [28]. Vascularized fibular graft is one of the technically difficult orthopedic surgeries which require technique of microvascular surgery. More recently, the addition of biological reagents has demonstrated positive results [30].

## Pure conservative therapy

A recent prospective, double-blind study showed that with conservative therapy using an oral bisphosphonate agent to prevent femoral head collapse, there was no difference in collapse progress between the placebo and treatment groups [31]. In Japan, novel clinical trials as a pure conservative therapy are performed and are registered to the public Japanese clinical trials registry, the University Hospital Medical Information Network (UMIN) Clinical Trials Registry. In recent years, bisphosphonates and parathyroid hormone have also started to be used to prevent collapse (UMIN000017582). In addition, for systemic lupus erythematosus patients undergoing steroid pulse therapy, a three-drug combination of the enzyme 3-hydroxy-3-methylglutaryl coenzyme A reductase inhibitor (pitavastatin), adenosine diphosphate receptor blocking antiplatelet (clopidogrel), and antioxidant (tocopherol) is co-administered (UMIN000008230). Other types of conservative treatment using an external device, such as hyperbaric oxygen therapy and extracorporeal shock wave lithotrity (UMIN000020197), have also been developed [32].

## Core decompression

**Fig. 2:**
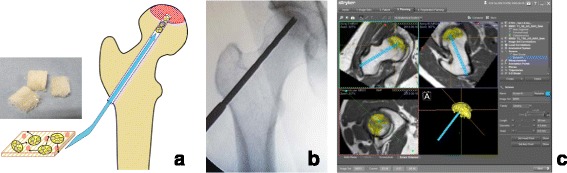
Regenerative therapy using controlled release of recombinant human fibroblast growth factor. Schematic views and photographs of the surgical procedure using recombinant human fibroblast growth factor (rhFGF)-2-impregnated gelatin hydrogel for patients with precollapse stage of osteonecrosis of the femoral head (ONFH) are shown. **a** A schema of the surgical procedure administering the rhFGF-2 gelatin hydrogel. The rhFGF-2-impregnated gelatin hydrogel is embedded percutaneously over the lateral aspect of the femur near the level of the lesser trochanter. A *small photograph* on the *left side* shows the actual gelatin hydrogel, which is a superior slow-release carrier for growth factors. **b** A representative intraoperative fluoroscopic image at drilling. **c** A screenshot of the preoperative planning using navigation software is shown. The *yellow area* shows the area of ONFH. The surgeon planned the suitable route of drilling (*blue screw*)

Core decompression is a minimally invasive surgery for ONFH dating back to the 1960s [28]. It was originally used to create a single large bone hole >10-mm diameter and had a high femoral head collapse rate. Over time, the method improved and now involves multiple bone holes of a smaller diameter (3 mm); this method has been popular since the 2000s. With the improved method, collapse rates are now 30 % for precollapse ONFH [33]. To further improve therapeutic efficacy to prevent femoral head collapse, cell transplantation, artificial bone, and growth factors have been used (Fig. 2).

## Regenerative therapy-based core decompression

### Metal implants

A preventive treatment involving the placement of cylindrical artifacts in the core decompression site has been developed in Europe and the USA. Such artifacts include implant rods made of Zimmer Trabecular Metal (porous tantalum, manufactured by Zimmer, Warsaw, IN, USA), which are available in both small and large diameters. Tsao et al. used a large-diameter rod in 113 joints from 97 patients with precollapse ONFH; over 4 years, 19 joints (19.6 %) underwent THA [34]. Similarly, Veillette et al. reported that of 48 joints from 42 patients, 16 (33.3 %) experienced a collapsed femoral head over 4 years [35]. In addition, Floerkemeier et al. reported that in 23 joints with ONFH, 13 (56.5 %) required THA after an average of 1.45 years. In contrast to other studies, the outcome after core decompression combined with tantalum rod insertion was not superior compared to core decompression alone [36].

### Artificial bones

Yu et al. reported that injectable calcium phosphate-based artificial bone (CaSO_4_/CaPO_4_ composite) was used alone for ONFH. In 19 joints from 18 patients, 3/6 joints (50 %) with precollapse ONFH and 8/13 joints (61.5 %) with early collapsed ONFH were reported to have undergone THA after an average of 8.5 months post-surgery [37].

### Cell transplantation

In ONFH, progression of the necrotic area and occurrence of additional necrotic areas are extremely rare [38]. Therefore, regeneration of the necrotic bone to normal bone tissue could make it possible to cure ONFH. To regenerate the necrotic bone, there is a need to promote remodeling and angiogenesis as well as absorption of the necrotic bone. However, clinical results for free bone grafts do not indicate significant improvement over core decompression. Thus, a new treatment strategy has been attempted in which cell transplantation, such as autologous bone marrow mononuclear cells, was employed to regenerate the necrotic bone directly. As reported by Hernigou and Beaujean, bone marrow was taken from the iliac crest, and the mononuclear cell-containing fraction was separated using a cell centrifugal separation device [12]. The cells were injected into the necrotic bone area from the core decompression site that promotes the remodeling of cancellous bone regeneration and necrosis. After 8–18 years, only 94 of the 534 patients (17.6 %) with precollapse ONFH were reported to have femoral head collapse [16]. In addition, Gangji et al. conducted a prospective double-blind study on 24 hips, in which core decompression and autologous bone marrow mononuclear cell transplantation combination were compared over 5 years. Progress and structural destruction of the subchondral bone were noted in 8 of the 11 joints (72.7 %) in the core decompression group and in 3 of the 13 joints (23.1 %) in the combination group [13]. Furthermore, in a systematic review, Papakostidis et al. reported that in the precollapse stage, core decompression with autologous bone marrow cell implantation into the femoral head is clinically effective and can improve survivorship of the femoral heads and reduce the need for THA [39]. Another systemic review by Lau et al. reported that cell therapy is considered a reliable regenerative approach to engraft more cells in the necrotic area and that further development of a method promoting differentiation is needed [40].

In combination with cell therapy, the use of artificial bone in the core decompression site has also been developed. For instance, Civinini et al. reported that, with a combination of bone marrow cell transplantation and artificial bone, 5/30 joints (16.6 %) with precollapse ONFH and 3/7 joints (42.8 %) with early collapsed ONFH further collapsed or progressed further an average of 20.6 months post-surgery [41]. Yamasaki et al. reported that 30 joints from 22 patients received transplanted bone marrow mononuclear cells with interconnected porous calcium hydroxyapatite into the femoral head; over an average of 29 months, 13 (43.3 %) experienced femoral head collapse [42].

Autologous bone marrow cell transplantation may be reliable in early-stage ONFH patients [12, 13, 16, 39, 40]. Ex vivo amplification of bone marrow cells has been proposed to increase the number of injected stem cells [13–15, 30].

### Growth factors

Bone regeneration in joint areas using cell growth factors, another treatment approach, has become an important topic of research. Problems with protein therapy include an extremely short half-life and side effects with systemic or high-dose administration. The use of rhFGF-2-impregnated gelatin hydrogel has the advantage of sustained release over rhFGF-2 solution because its biologic half-life period is short [21]. A slow-release system built around a bioabsorbable gelatin hydrogel enables local administration with excellent controlled release, greatly contributing to establishing practical use for protein therapy. Such factors useful for bone regeneration include transforming growth factor-β, bone morphogenetic protein (BMP), FGF-2, vascular endothelial growth factor, and insulin-like growth factor [2, 3, 9]. For ONFH, the use of BMP in combination with bone grafts has been reported. For example, Lieberman et al. co-administered rhBMP (50 mg) with an allogeneic fibular graft after core decompression. Of 16 cases with precollapse ONFH, two (12.5 %) collapsed after an average of 53 months [17]. Moreover, Papanagiotou et al. co-administered rhBMP 3.5 mg with an autologous fibular graft; of five cases with precollapse ONFH, one (20 %) experienced femoral head collapse over an average of 4 years [43].

In the field of bone and joint medicine, the angiogenic and osteogenic actions of rhFGF-2 have been the subject of numerous reports [21, 23, 24, 44, 45]. In particular, the use of gelatin hydrogel as a superior slow-release carrier has produced increased bone mass in areas of bone deficit [21]. Furthermore, rhFGF-2 administration produced rapid osteogenesis and increased bone mass in humans with lower leg fracture [44] and osteotomy surface [45]. A strategy using growth factors has also been proposed to treat ONFH. We reported the first clinical application of rhFGF-2-impregnated gelatin hydrogel for patients with precollapse stage of ONFH (Fig. 2) [24]. Ten patients with femoral heads up to precollapse stage 2 underwent a single local administration of 800-μg rhFGF-2-impregnated gelatin hydrogel and followed up for 1 year. Primary outcomes included adverse events and complications. Secondary outcomes included changes in Harris Hip Scores, visual analog scale pain scores, and UCLA activity rating scores, radiological changes as determined via radiographs, computed tomography scans, and MRI of the hip joint. One-year short-term results of this pilot study indicated the approach was safe and feasible. During 1-year follow-up of 10 patients, there was only one case of femoral head collapse; however, this occurred in a hip with extensive necrosis. Stage progression and collapse did not occur in the other nine cases. The results of previous joint-preserving regenerative therapy-based core decompression are presented in Table 1.

**Table 1:** Joint-preserving regenerative therapy-based core decompression

First author year/design	Technique	Number of hips (precollapse)	Background factors for ONFH (%)	Mean age (years)	Mean follow-up (years)	Hip survivorship (%)
Tsao [34] 2005/P	CD	94	S 41, A 24, I 30, others 5	43	4.0	80.4
	TR					
Veillette [35] 2006/R	CD	50	S 45, A 3, I 26, T 10, others 16	35	4.0	66.7
	TR					
Floerkemeier [36] 2011/P	CD	23	NR	40	1.4	43.5
	TR					
Yu [37] 2015/P	CD	6	S 5, A 68, I 21, T 5	48	1.4	50.0
	CaSO_4_/CaPO_4_					
Hernigou [16] 2009/P	CD	534	S 19, SCD 31, I 28	39	13.0	82.4
	BMMNC					
Gangji [13] 2011/RCT	CD	11	S 82, A 9, I 9	45.7	5.0	27.3
	ᅟ					
Gangji [13] 2011/RCT	CD	13	S 85, A 8, I 8	42.2	5.0	76.9
	BMMNC					
Civinini [41] 2012/P	CD, BMC,	30	S 49, A 35, I 16	43.9	1.7	83.3
	CaSO_4_/CaPO_4_					
Yamasaki [42] 2010/R	CD	9	S 22, A 44, I 33	49	2.4	0
	HA					
Yamasaki [42] 2010/R	CD, BMMNC,	27	S 73, A 20, I 7	41	2.4	56.7
	HA					
Lieberman [17] 2004/P	CD, FBG	16	S 76, A 18, S&A 6	47	4.4	87.5
	rhBMP 50 mg					
Papanagiotou [43] 2014/P	CD, FBG	5	S 40, A 20, I 40	32	4.0	80.0
	rhBMP 3.5 g					
Kuroda [23] 2015/P	CD	10	S 80, A 20	39.8	1.0	90.0
	rhFGF-2 800 μg					

*P* prospective study, *R* retrospective study, *RCT* randomized clinical trial, *CD* core decompression, *TR* tantalum rod, *BMMNC* bone marrow mononuclear cell, *BMC* bone marrow cell, *HA* hydroxyapatite, *FBG* fibular bone graft, *rhBMP* recombinant human bone morphogenetic protein, *ONFH* osteonecrosis of the femoral head, *S* steroid use, *A* alcohol intake, *I* idiopathic, *T* trauma, *NR* not reported, *SCD* sickle cell disease

**Fig. 3:**
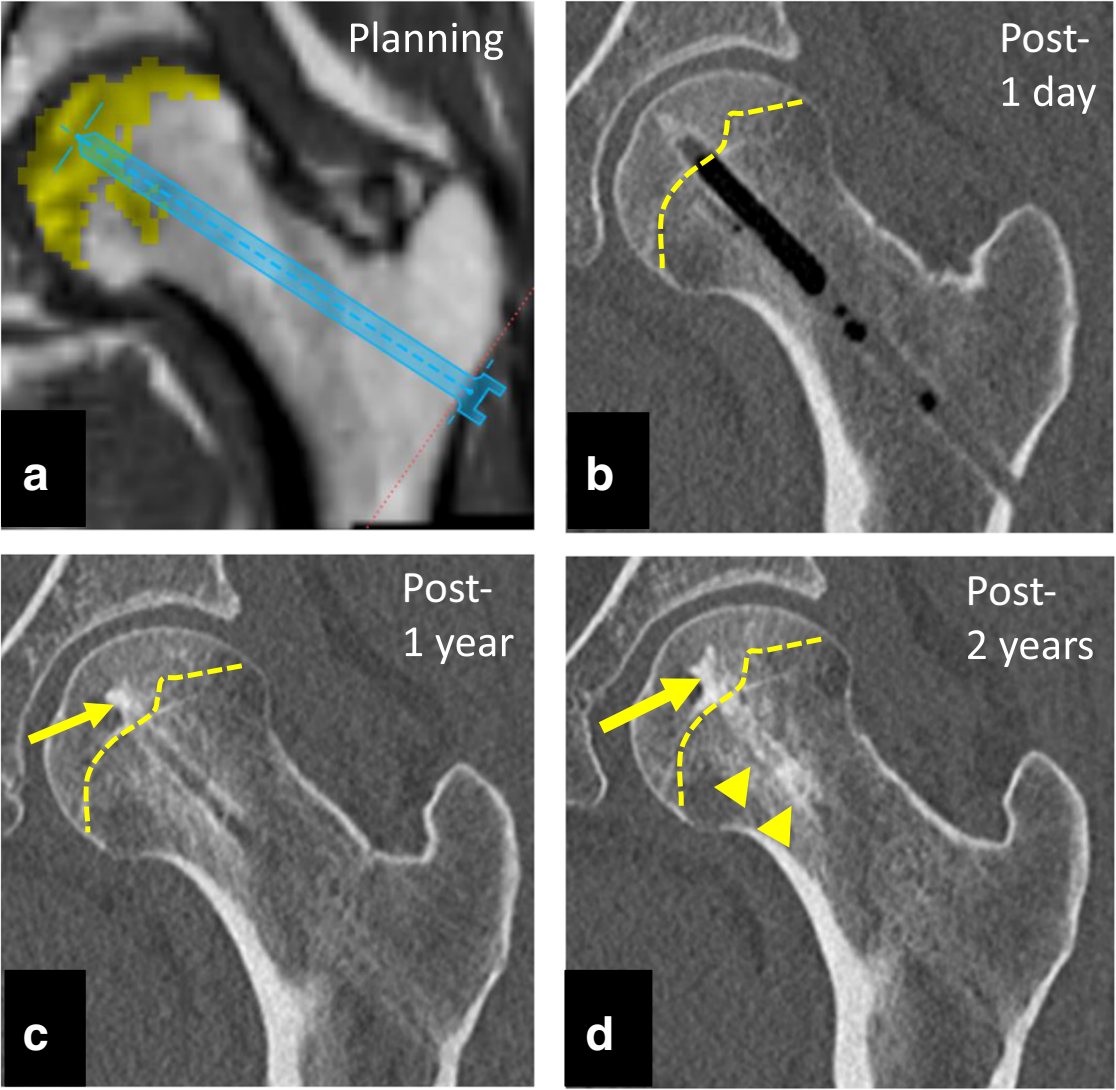
Planning and representative computed tomography images. **a** A screenshot of the preoperative planning. **b** Coronal computed tomography image shows a bone defect at the drilling route and implanted region 1 day postoperatively. The *yellow dashed line* shows the border of the osteonecrotic area of the femoral head. **c** In contrast, apparent bone regeneration of the osteonecrotic area is observed at 1 year postoperatively (*yellow arrow*). The normal contour of the femoral head is maintained. **d** Apparent bone regeneration of the osteonecrotic area is observed in the implanted region (*yellow arrow*) and drilling route (*yellow arrowheads*) at 2 years postoperatively. Normal contour, thick trabecular bone, and bone regeneration of the drilling route can be observed

**Fig. 4:**
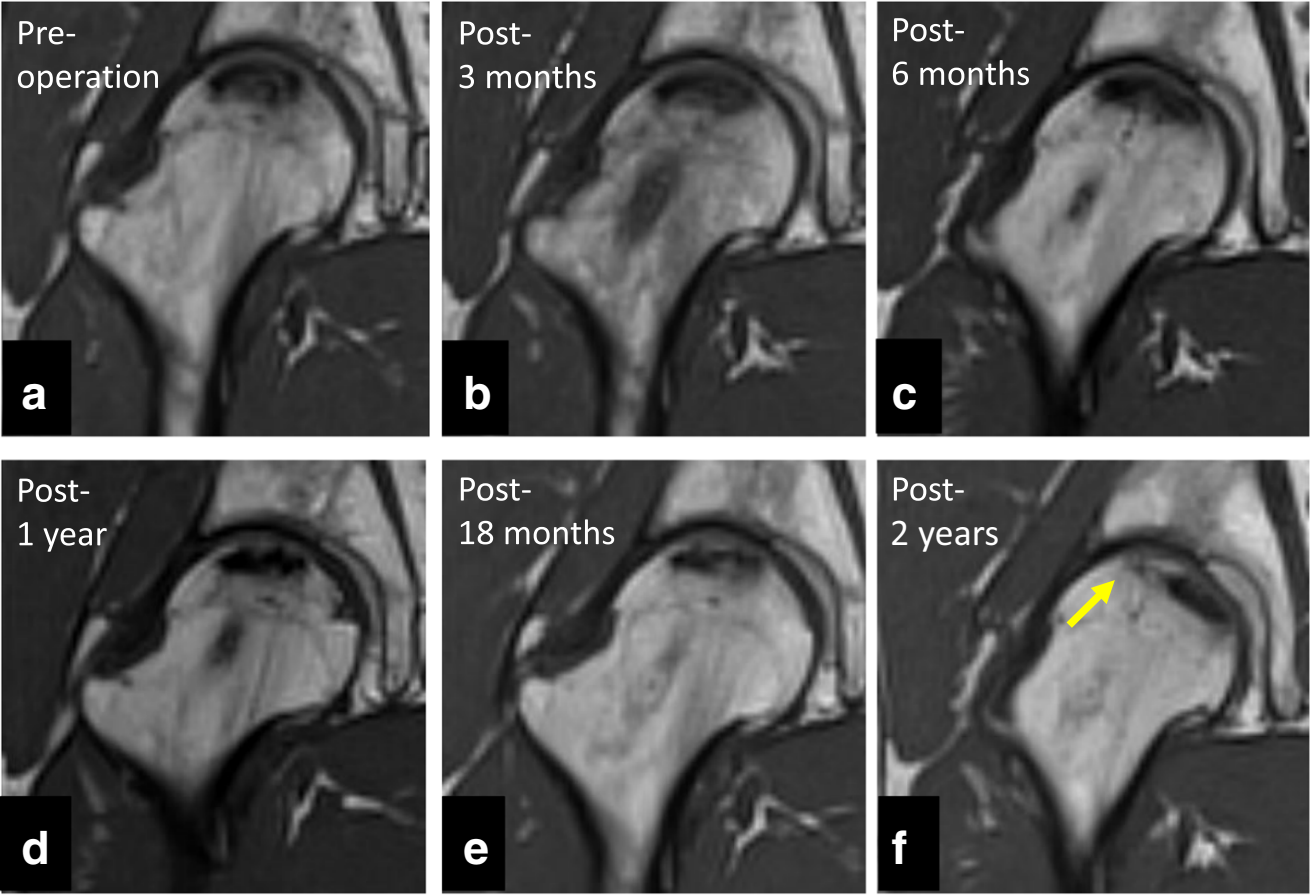
Representative magnetic resonance images. **a** Preoperative coronal T1-weighted magnetic resonance imaging (MRI) showing osteonecrosis of the femoral head (ONFH) that occupied the weight-bearing portion and extended laterally to the acetabular edge. **b**–**d** MRI scan of the ONFH area and the femoral neck region 6 months and 1 year postoperatively, showing continued low signal intensity, indicating the influence of the traumatic procedure. **e** MRI scan 18 months postoperatively, showing the first change of signal intensity at the drilling route. The drilling site at the femoral neck is changing to the normal signal intensity of the bone. **f** Most recent MRI scan 2 years postoperatively, showing almost normal signal intensity at the ONFH area. The area and size of ONFH decreased at the weight-bearing surface (*yellow arrow*)

The minimally invasive therapy (1-cm skin incision) attempted to prevent femoral head collapse through direct administration of rhFGF-2, which has both angiogenic and osteogenic actions. Computed tomography (Fig. 3) and recent MRI (Fig. 4) confirmed bone regeneration and reduction of the necrotic area. Additionally, hospitalization costs were dramatically reduced to 10 % of that for THA. From an economic standpoint, avoiding the need for artificial joints could greatly reduce medical expenses. However, it is still unclear whether rhFGF-2 administration or conservative treatment has a better efficacy. Further studies with longer follow-up are needed to analyze and evaluate the survival rates of femoral heads treated with rhFGF-2 administration or conservative treatment.

## Conclusions

The most recent regenerative therapy for ONFH has aimed to induce bone regeneration to prevent femoral head collapse. To develop the practical clinical application for regenerative ONFH therapies, suitable sources, including cell sources, artificial materials, specific proteins, or combinations thereof, must be identified. With the use of various regenerative therapies, including cell therapy, implants, and recombinant growth factors, the treatment for ONFH will reach a turning point in the near future.

### Consent for publication

This study was performed in accordance with the declaration of Helsinki. The entire treatment protocol was approved by the Ethics Committee of Kyoto University Graduate School and Faculty of Medicine. Written informed consent was obtained from all participants involved in the study. The clinical study reported herein was registered to the public Japanese clinical trials registry (UMIN000009250).

## Abbreviations

BMP: bone morphogenetic protein
MRI: magnetic resonance imaging
OA: osteoarthritis
ONFH: osteonecrosis of the femoral head
rhFGF: recombinant human fibroblast growth factor
THA: total hip arthroplasty
